# Role of infectious agents in the carcinogenesis of brain and head and neck cancers

**DOI:** 10.1186/1750-9378-8-7

**Published:** 2013-02-02

**Authors:** Kenneth Alibek, Ainur Kakpenova, Yeldar Baiken

**Affiliations:** 1 Nazarbayev University, 53 Kabanbay Batyr Avenue, Astana, 010000, Kazakhstan; 2 Republican Scientific Center for Emergency Care, 3 Kerey and Zhanibek Khan Street, Astana, 010000, Kazakhstan

**Keywords:** Carcinogenesis, Brain cancer, Head and neck cancer, Cytomegalovirus, Polyomavirus, Toxoplasma, Epstein-Barr virus, Human papilloma virus, HIV, Streptococcus anginosus

## Abstract

This review concentrates on tumours that are anatomically localised in head and neck regions. Brain cancers and head and neck cancers together account for more than 873,000 cases annually worldwide, with an increasing incidence each year. With poor survival rates at late stages, brain and head and neck cancers represent serious conditions. Carcinogenesis is a multi-step process and the role of infectious agents in this progression has not been fully identified. A major problem with such research is that the role of many infectious agents may be underestimated due to the lack of or inconsistency in experimental data obtained globally. In the case of brain cancer, no infection has been accepted as directly oncogenic, although a number of viruses and parasites are associated with the malignancy. Our analysis of the literature showed the presence of human cytomegalovirus (HCMV) in distinct types of brain tumour, namely glioblastoma multiforme (GBM) and medulloblastoma. In particular, there are reports of viral protein in up to 100% of GBM specimens. Several epidemiological studies reported associations of brain cancer and toxoplasmosis seropositivity. In head and neck cancers, there is a distinct correlation between Epstein-Barr virus (EBV) and nasopharyngeal carcinoma (NPC). Considering that almost every undifferentiated NPC is EBV-positive, virus titer levels can be measured to screen high-risk populations. In addition there is an apparent association between human papilloma virus (HPV) and head and neck squamous cell carcinoma (HNSCC); specifically, 26% of HNSCCs are positive for HPV. HPV type 16 was the most common type detected in HNSCCs (90%) and its dominance is even greater than that reported in cervical carcinoma. Although there are many studies showing an association of infectious agents with cancer, with various levels of involvement and either a direct or indirect causative effect, there is a scarcity of articles covering the role of infection in carcinogenesis of brain and head and neck cancers. We review recent studies on the infectious origin of these cancers and present our current understanding of carcinogenic mechanisms, thereby providing possible novel approaches to cancer treatment.

## Introduction

More than 237,000 people are diagnosed with brain cancer annually [1]. Central nervous system (CNS) tumours are classified by the World Health Organization according to histological pattern, cell behaviour, and cytogenetics. A significant proportion is neuroepithelial tumours, which include glial and non-glial tumours. Classification additionally includes tumours in the meninges (meningioma); germ cell tumours; tumours of the sellar region, CNS lymphatic tissue, and peripheral nerves; and metastatic tumours. Histological features and malignancy of the tumour are graded from Grade I to Grade IV for uniformity and prognostic purposes [2].

Each year, 500,000 new head and neck cancer cases are diagnosed worldwide [3]. The classification of these tumours is based mostly on histological and clinical findings [4,5]. Most head and neck cancers are squamous cell carcinomas progressing from the thin epithelial lining of the head and neck tissue.

Although many potential risk factors for brain and head and neck cancers have been identified, including smoking and chewing tobacco, alcohol consumption, poor diet combined with a hypodynamic lifestyle, acid reflux disease, haematopoetic stem cell transplantation, ionising radiation, electromagnetic fields, and exposure to carcinogenic chemicals, various pathogenic infections also constitute an under-appreciated but significant risk [6,7]. The International Agency for Research on Cancer (IARC) estimated that 16% of total new cancer cases and 20% of deaths from cancer worldwide in 2008 were due to infections. Infectious agents are thought to cause various pathological alterations including DNA mutations, cell cycle modulation, dysregulation of DNA repair mechanisms, chronic inflammation, and immune system impairment [8-13]. Therefore, treating such conditions and their cause (i.e., infections) may interfere with carcinogenesis or even prevent cancer. This approach has been approved worldwide for *Helicobacter pylori*, hepatitis B and C viruses, and human papillomaviruses, and shown to prevent corresponding cancers such as gastric, liver, and cervical cancer. This review concentrates on recent updates on brain and head and neck oncological diseases, which are combined for analysis due to their common localisation in the head region. These cancers are also associated with a number of pathogens although it is not yet clear whether the infectious agents actually cause the cancer or act as co-factors or bystanders. So, the mechanisms underlying precancerous conditions and carcinogenesis associated with infections will be discussed.

### Brain cancer

Brain cancers include tumours located within the brain and CNS. Among neuroepithelial tumours, the most frequent (50-60%) is glioblastoma. Glioblastoma multiforme (GBM) is highly anaplastic and develops from a diffuse astrocytoma or *de novo*[2]. GBM is often found in the cerebral hemispheres and its peak incidence occurs at an age of 45–70 years. Some tumours occur more frequently in children, such as medulloblastoma, brain stem glioma, ependydoma, and pineal tumour. Medulloblastoma is often found in the cerebellum and can spread to other CNS regions. Histopathology indicates that medulloblastoma originates from primitive undeveloped cells. Some brain tumours can be benign, such as meningiomas arising from membranes of the brain and CNS [2].

### Glioblastoma and cytomegalovirus

The role of human cytomegalovirus (HCMV) in the pathogenicity of glial tumours is attracting increasing interest. CMV DNA or antigen levels are elevated in many cancer types, and CMV is directly detected at a high frequency in brain cancers. Using highly sensitive detection techniques such as immunohistochemical detection and PCR amplification, HCMV nucleic acids and genes were found in >90-95% of GBM tumours [14] and CMV proteins pp65 or IE1 were detected in approximately 50% of GBMs [15]. Markedly, the expression of IE1, pp65, and late antigens was detected in 100% of 27 GBM specimens, but not within surrounding brain tissue or other brain pathology specimens [16-19]. However, in a different study no circulating CMV was detected in peripheral blood of five GBM patients, possibly due to a subtype difference [20]. Moreover, it should be noted that glioblastoma incidence and cytomegalovirus seropositivity differs among ethnic groups [21].

### Medulloblastoma and cytomegalovirus

HCMV proteins were also detected in human medulloblastoma cell lines, accounting for as much as 92% of immediate early protein and 73% of late protein. In addition, a high level of CMV DNA and viral protein was identified in primary medulloblastoma, medulloblastoma cell lines, and xenografts [22]. Together, these findings from cell or tissue culture and patient blood analysis indicate a role of CMV in various types of brain cancer. However, further research on the underlying mechanisms is needed, as discussed further.

### Glial tumours, medulloblastoma, CNS tumours, and polyomaviruses

Brain cancer is commonly associated with polyomaviruses such as SV40, BKV, and JCV [23]. Association of SV40 with the development of brain tumours was first noticed in infected scientists who had worked with virus cultures [24]. Direct induction of SV40 oncogenesis was subsequently experimentally proved in many murine models [25]. Although some papers have reported BKV-associated central nervous system tumours [26,27], conflicting reports show no association [28]. Expression of JCV proteins and nucleic acids was detected in several cases of glial tumours, medulloblastoma, and lymphomas that localised to the nervous system. JCV infection can cause neurodegenerative abnormalities in the central nervous system (CNS), for example progressive multifocal leukoencephalopathy, which can be lethal [9]. Mechanisms that are common to all polyomaviruses are discussed further.

### Brain cancer and intracellular parasites

Association of brain cancers and neurocysticercosis with infection has been reported in several reviews [29,30] and is under continued investigation. Certain geographical associations have provoked interest in further research on this topic. Recent work by Thomas et al. (2011) showed that the incidence of brain tumours is higher in geographic regions where the protozoan parasite *Toxoplasma gondii* is common [31]. Another study revealed that brain cancer mortality increased with increased *T. gondii* seroprevalence in France [7]. Direct observation in experimental animals showed that *T. gondi* infection caused glioma. Regarding human specimens, *T. gondi* antibodies were found in astrocytoma [32] and meningioma [33] samples. Such associations and the direct presence of infectious particles in brain tumours warrant further research at the molecular level into the role of parasitic infection in carcinogenesis or the creation of a precancerous environment.

### Brain cancer and bacteria

Interestingly, there is not much information on the association of brain cancer with bacterial infection. *Mycoplasma* infections have been found in different types of carcinoma tissue, including glioma [34], however such infections are more commonly associated with cytokine-mediated damage and inflammatory lesions [35] leading to various CNS diseases [36]. Although one study reported that 5 of 20 medulloblastoma tumours contained *Brucella* DNA identified by the OMP31 primer/probe set, the association of neurobrucellosis with medulloblastoma remains uncertain and needs further study [37]. At the present time the low numbers do not support the association and the authors suspect that the DNA came from the diet rather than from infections. Although some bacterial species are suspected to be present in carcinoma tissues, there is currently insufficient information to conclude that bacteria play any role in brain cancer.

### Head and neck cancers

Biologically, head and neck cancer refers to the group of cancers located in the aerodigestive tract including lip, oral cavity, nasal cavity, paranasal sinuses, pharynx and larynx, oropharynx and hypopharynx, as well as the salivary glands and local lymph nodes. Most cases of head and neck cancer (approximately 90%) are squamous cell carcinomas. Head and neck squamous cell carcinoma (HNSCC) is derived from the mucosal lining throughout the local region, inducing tumour development in the nasal and oral cavities, nasopharynx, larynx, oropharynx, hypopharynx, and paranasal sinuses.

### Nasopharyngeal carcinoma and EBV

Epstein-Barr virus (EBV) has classically been associated with nasopharyngeal carcinoma (NPC), Burkitt’s lymphoma, and Hodgkin’s lymphoma and, to lesser extent, HIV-positive CNS lymphomas and hypopharyngeal and laryngeal tumours [38]. NPC is a head and neck cancer that occurs endemically in some countries of the Mediterranean region and Asia, where EBV antibody titers can be measured to screen high-risk populations. It is also a leading cancer of the epithelial cell lining, and is responsible for nasopharyngeal neoplasms that are common in adolescents and children. All types of NPC occur twice as frequently in males than in females, and type 2 and 3 carcinomas are associated with EBV virus titre levels [39,40]. Almost every undifferentiated NPC is EBV-positive, irrespective of geographical origin [41]. The EBV virus was classified as a group 1 carcinogenic agent by the IARC.

### HNSCC and human papillomavirus (HPV)

It is established that HPV types 16, 18, 31, and 45 play a significant role in cervical cancer, as well as anal, vaginal, and other anogenital cancers. Recent studies showed that certain types of HPV are also closely related with malignant growth of the epithelial mucosa lining in head and neck areas. HPV-related tumours display clinical and molecular characteristics that are distinct from HPV-unrelated tumours such as those caused by alcohol consumption and tobacco smoking. HPV-associated HNSCC seems to have a better prognosis than HPV-negative tumours due to increased sensitivity to chemoradiotherapy. In particular, 90% of HPV-positive HNSCCs are positive for HPV type 16 [42], and the dominance of HPV type 16 in HPV-positive HNSCC is even greater than that seen in cervical carcinoma (50%-60%). The high-risk subtypes of HPV involved in cervical carcinogenesis have shown similar associations with HNSCC [43]. Moreover, an increasing number of HPV-associated oropharyngeal cancers has been noted during the last few decades worldwide and has been linked to sexual behaviour [42].

### Kaposi’s sarcoma and HIV/HHV-8

An association of HIV with head and neck cancers was revealed back in the early 1980s along with the first clinical observation of its association with symptoms such as lymph node hyperplasias, candidiasis, herpes virus infections, and neoplasias such as Kaposi’s sarcoma (KS) or HIV-associated Non-Hodgkin’s lymphomas (HIV-NHL) that were previously recognised as common ear, nose, and throat (ENT) disorders [44]. The most common HIV-associated malignancy is Kaposi’s sarcoma, accounting for up to 80% of HIV-related cancers [45]. KS is normally a rare type of cancer, but after HIV-1 infection its incidence is greatly elevated, reaching a 70,000-fold increased incidence in HIV-infected homosexual men [46]. The oral cavity is the most common site of otolaryngological manifestations, and nearly 95% of lesions are found on the palate. Other sites include gingival surfaces of the oropharynx, external ear, larynx, and nose [47]. Endemic KS and AIDS-associated KS (AIDS-KS) tend to be more aggressive than classical KS and AIDS-KS lesions often rapidly progress to plaques and nodules affecting the upper trunk, face, and oral mucosa.

Human herpes virus type 8 (HHV-8) [or Kaposi’s sarcoma-associated herpes virus (KSHV)] was originally identified as a human oncogenic herpes virus [48] expressing candidate viral oncogenes that constitutively activate growth-signalling pathways [49,50] and has subsequently been shown to have a strong correlation with different forms of KS. HHV-8 is predominantly found in tumour spindle cells but has also been detected in lymphocytes, monocytes, and keratinocytes [49,51].

### Oral squamous cell carcinoma (OSCC) and bacterial and mycotic infections

There is an apparent link between some bacterial species and certain forms of cancer. In addition to tobacco and alcohol consumption, *S. anginosus* infection is a significant risk factor for eosophageal and head and neck cancer, precancerous leukoplakia, and squamous cell carcinoma, although the exact mechanisms are unknown [6,52,53]. One report [6] indicated positivity for *S. anginosus* DNA in samples obtained from head and neck cancers by PCR and Southern blot analyses (100% and 33% respectively). *S. anginosus* DNA was detected more frequently in squamous cell carcinoma than other forms of cancer and showed a correlation with aerodigestive tract cancer [52,54].

Other bacterial pathogens such as *Prevotella melaninogenica, Eubacterium saburreum, C. gingivalis, Leptotrichia buccalis,* and *Streptococcus mitis* species demonstrated higher levels of accumulation in OSCC patients than in control groups [55]. It has been proposed that alteration of tumour cell receptors could change the adhesion of certain bacterial species [56].

*Exiguobacterium oxidotolerans, P. melaninogenica, Staphylococcus aureus, Veillonella parvula,* and *Micrococcus* species were isolated from OSCC sites but not from control sites, whereas *Moraxella osloensis, P. vevoralis,* and *Acromyces* were found only in control samples. The majority of microorganisms isolated from tumours were saccharolytic and acid-tolerant, such as yeasts, actinomycetes, bifidobacteria, lactobacilli, streptococci, and *Veillonella*, indicating an acidic and hypoxic tumour microenvironment.

Fungal infections such as chronic hyperplastic candidosis caused by *Candida* species showed association with the invasion of candidal hyphae into oral epithelium and progression to dysplasic changes [55].

### Mechanisms of carcinogenesis

#### Mechanisms in brain cancers

Consensus was recently reached on the oncomodulatory role of HCMV in gliomas when HCMV was shown to interact with the key signalling pathways in cancer development [57]. The mechanism of glioblastoma development was recently elucidated by Bleeker et al., who showed that many of the alterations involve PI3K/AKT, retinoblastoma (Rb), and p53, signalling pathways downstream of the tyrosine kinase receptor [58]. Interestingly, CMV-infected cells also exhibit reduced p53 and Rb function. CMV influences carcinogenesis not only by expression of cell proliferation factors, but also by immunosuppression [8]. CMV interleukin (IL)-10 was shown to convert monocytes and microglia in glioblastomas to an immunosuppressive phenotype that supports tumour development [18]. In immunocompetent individuals CMV infection is asymptomatic, but constant antigenic pressure leads to an increase in CD8 + CD57+, CD4 + CD28-, and CD8 + CD28- cells specific to CMV antigen pp65. These cells express enkephalin-inhibiting receptors, thus blocking the functions of cytotoxic lymphocytes [9]. In addition, analysis of CMV proteins showed that US28 is a constitutively active chemokine receptor that induces an oncogenic phenotype by increasing the expression of COX-2, PGE2, and IL6 [59].

A characteristic feature of JCV infection is early activation of the human neurotropic viral promoter JCVE, which initiates transcription of large T-antigen [60,61]. The major molecular mechanisms of polyomavirus oncogenesis involve inhibition of tumour suppressors by viral T-antigens and epigenetic transcriptional regulation through histone acetylation. T-antigens of all papovaviruses bind to Rb, leading to disruption of cell cycle regulation. They also bind to and inactivate CBP/p300 and p53 proteins. Large T-antigen shows mutagenic potential towards cellular DNA and prevents DNA repair. Less is understood about the oncogenic properties of small T-antigen, but it is known to play a mitogenic role and is involved in cell transformation and uncontrolled cell proliferation [9,61]. Other viral proteins (e.g., agnoprotein) bind to p53 and increase the activity of p21/WAF-1 [62].

The effect of parasites on host immunity increases the risk of brain cancer by triggering chronic inflammation, inhibiting apoptosis, and through transfer of genetic material from the parasite to the host [31]. For example, *T. gondii* persists in human pseudocysts, macrophages, and neurons. In the latent phase the parasite is in the bradyzoite stage and causes mild inflammation as a result of the host’s immune response [63]. However, dying cysts probably cause intense inflammation. In the case of *Taenia solium*[64] and *Plasmodium*[65], persistent cysts trigger the mitogenic response of lymphocytes to achieve immunosuppression. In addition, cysticerci of *T. solium* initially stimulate the immune response to produce a protein source for the parasite and subsequently acquire the host membrane proteins thus avoiding immunodetection. Larvae of *Schistosomula* resemble native lymphocytes and evade the immune response [29]. Summing up, any interference with, or evasion of, the host immune system may predispose an organism to cancer, although much remains to be learned about the interactions between parasites, the host immune response, and brain cancer development.

### Mechanisms in head and neck cancers

In EBV-associated carcinogenesis, the virus infects B lymphocytes through binding of the major envelope glycoprotein gp350 to the B cell CD21 receptor and binding of a second glycoprotein, gp42, to MHC class II molecules as a co-receptor [10]. The transformation of EBV viral particles [Epstein-Barr nuclear antigens (EBNAs), latent membrane proteins (LMPs) and Epstein-Barr virus-encoded small RNA (EBERs)] from B cells into latently infected lymphoblastoid cell lines, both in lytic and latent cycles, ultimately affects host genome regulation causing dysregulation of apoptosis, genetic instability, and constant proliferation. It has also been suggested that EBV enters nasopharyngeal cells through IgA-mediated endocytosis. Because the EBV genome is monoclonal in nature, EBV infection occurs before clonal expansion of the malignant cells [10,41].

Recent studies showed that non-polyadenylated RNAs (EBERs) are present in all infected cells. This supports the idea that EBV infection is involved in an early stage of NPC carcinogenesis [10]. Molecular abnormalities in NPCs are complex and include inactivation of RASSF1A and p16 tumour suppressor genes on 3p21 and 9p21 chromosomal regions by homologous deletion and promoter methylation during low-grade dysplasia stages. These events occur early in pathogenesis and might predispose the abnormal epithelium to subsequent infection with EBV derived from adjacent lymphoid tissues and circulating B-cells. Inactivation of TSCL1, EDNRB, and death-associated protein kinase genes via promoter methylation is also frequently observed. Gene expression profiling showed dysregulation of PI3K/Akt, Wnt/β-catenin, TGF-8, and mitogen-activated protein kinase (MAPK) signalling pathways in NPC with upregulation of NF-k82, survivin, and Bcl-2, nuclear accumulation of β-catenin, and dysregulation of integrins [10]. Almost all EBV genes are expressed in the active phase of infection. The early genes BRLF1 and BZLF1, which code transcriptional transactivator proteins that promote cell replication, induce expression of the viral genes. BZLF1 and BRLF1 proteins also inhibit the tumour suppressors p53 and pRb, respectively [9].

HPV-related carcinogenesis involves integration of the HPV DNA into the host cell and expression of the viral oncoproteins E6 and E7. E6 protein induces degradation of p53 through ubiquitin-mediated proteolysis, leading to loss of p53 activity. E7 protein binds to pRb, inducing S-phase entry and leading to proliferation, malignant transformation, and cell-cycle dysregulation. E6 and E7 proteins can activate expression of somatic cell telomerases, inducing proliferation and cellular division. In humans, 85% of malignant neoplasias show elevated telomerase activity, compared with only 27% of benign tumours. Telomerase activity in HPV-infected cells decreases the expression of pRb and p53 and increases expression of p21WAF1 and p16INK4a *in vitro*. Moreover, p21WAF1 is inactivated, probably due to binding of E7 protein. These alterations lead to loss of control of the G2/M transition and result in accumulation of mutations and instability of the affected cell’s genome [9].

The pathogenesis process of KS appears to be complex, involving various mechanisms dependent on both viral and cellular activities, and is related to inflammation and angiogenesis promoted by endothelial growth factors (β-FGF, PDGF, VEGF) and HIV-Tat as well as cell proliferation and anti-apoptotic activity through vBCL2 [49,66,67]. The role of HIV-1 infection in the initiation and progression of AIDS-KS involves two crucial paracrine mechanisms: production of the HIV Tat protein and promotion of cytokine production [68]. HIV-1 Tat is an 86-amino acid protein with the primary function of increasing the processivity of RNA polymerase II and transcription initiation [69,70]. In contrast, there is no clear evidence for the role of HHV-8 in KS pathogenesis. Based on current data, HHV-8 infection appears to be necessary, but not sufficient, for the development of KS and additional co-factors, such as hormones, genetic predisposition, and/or co-infection with other infectious agents, are probably required for disease development [71].

Several mechanisms have been proposed for bacterial carcinogenesis. Studies have indicated that several bacteria can cause chronic infections by producing toxins that interfere with the cell cycle and lead to changes in cell growth [72-74]. Other chronic bacterial infections induce cell proliferation via activation of MAPK pathways and cyclin D1 [75], and several infections can suppress apoptosis by modulation of Bcl-2 proteins or by inactivation of pRb [76]. Also, many pathogenic bacteria that cause chronic infection with intracellular access destroy the host cell signalling pathways, thus enhancing survival of the pathogen [77]. Regulation of these signalling factors is essential for the development or inhibition of carcinogenesis. Such infections can mimic some of the major events in tumour development, and indeed the precancerous lesions formed in such infections can regress with antibiotic treatment and elimination of bacteria.

### Concluding notes: Can carcinogenesis be inhibited by treatment against infections?

Various studies have shown that anti-viral and anti-bacterial drug treatments have a favourable effect on prognosis through prevention of tumorigenesis. Thus, oncologists may benefit from understanding the mechanisms of infection-related carcinogenesis to develop novel cancer treatment strategies. A summary of this information is presented in Table 1.

**Table 1:** Cancer diseases and associated infectious agents

***Type of tumour***	***Infectious Agent***	Direct/indirect carcinogenesis*	***Mechanism of Carcinogenesis***
Glioblastoma	CMV	indirect	Interference with signaling pathways, reduced p53 and Rb function, increasing level of telomerase, angiogenic factors, cell motility and invasion. Chronic inflammation, expression of COX-2, PGE2, VEGF, IL-6 production, STAT3 phosphorylation [8,58,59]
Medulloblastoma			
Glioma	JC	direct	Viruses express T-antigen and agnoprotein, which binds to tumour supressors p53 and Rb, inactivate CBP/p300, increase the activity of p21/WAF-1, prevents DNA repair; small T-antigen involved in cell transformation;cause chromosomal aberrations [9,60-62]
Medulloblastoma			
Multifocal leukoencephalopathy			
Neuroblastoma	BK	direct	
Meningioma	SV40	direct	
Glioma	*Toxoplasma gondii*	indirect	Chronic inflammation, mutation accumulation, inhibit apoptosis; cysts trigger the mitogenic response of lymphocytes to achieve immunosuppression [63]
Astrocytoma			
Meningioma			
Glioma	*Mycoplasma*	indirect	Cytokine-mediated damage and inflammatory lesions [34-36]
CNS lymphoma	EBV	direct	Transformation of B-cells [78]
Nasopharyngeal carcinoma	EBV	direct	RASSF1A promoter methylation, p16 homozygous deletions and methylation [9,10,41]
Burkitt’s lymphoma			
Hodkin’s lymphoma			
HNSCC	HPV	direct	Loss of p53 and pRb, activation of telomerase [9]
Kaposi’s Sarcoma	HIV/HHV-8	direct	Angiogenesis promoted by endothelial growth factors (β-FGF, PDGF, VEGF) and HIV-Tat as well as cell proliferation and anti-apoptotic activity through vBCL2 [49,66-71]
HIV-NHL			
Eosophageal cancer	*S. anginosus*	indirect	Mechanism needs further investigation
HNSCC			
OSCC			
HNSCC	*Candida spp.*	indirect	Similar ability to promote neoplastic changes as the known promoter phorbol-12,13-didecanoate [79]
OSCC			

*Agents Classified by the *IARC Monographs*, Volumes 1–106 [80].

Recently developed vaccines designed to prevent infection with HPV types 16 and 18 include Cervarix and Gardasil, which have proved effective against cervical cancer several years after vaccination [81]. As an example of multivalent vaccine prevention, recent preclinical studies show that vaccination against HPV 16, 18, 31, 45, and 52 subtypes targeting E7 proteins in mammals (e.g., mice and Rhesus monkeys) result in anti-tumour responses and reduction of the TC-1 tumour cell population [82,83]. The recombinant protein-based vaccine Pentarix elicits a CD8 response against five strains of HPV in human. These findings suggest that prevention of infection with HPV subtypes is crucial for inhibiting tumour development in HPV-associated malignant neoplasia. Another study involving synthetic vaccine against high-risk HPV type 16 demonstrated induction of a potent T-cell response and complete regression of all lesions and eradication of virus in 9 of 20 women with high-grade vulvar intraepithelial neoplasia [84]. These therapeutic vaccines can therefore trigger a sufficient immune response to target the pathogen and suppress cancer development.

CMV is detectable in the majority of GBMs and could be a potential target for treatment. Even low levels of CMV gene expression can be used for immunologic targeting [18]. Interestingly, T-cell therapy for patients with CMV + GBMs was very beneficial and has entered phase I/II clinical trials [85]. Such therapies expand CMV-specific T-cell populations, which recognise pp65+ and IE1+ targets and kill CMV-infected autologous GBM cells [85].

Similarly, in EBV-related nasopharyngeal carcinoma, treatment of the infection produced promising outcomes with respect to cancer prevention. Yoshizaki et al. [86] demonstrated that treatment of EBV by injection of the anti-viral drug cidoflovir into the tumour once every 3 weeks suppressed tumour growth, and analysis of EBV-encoded RNA revealed reduction within the tumour cell population. In addition, several CNS diseases including meningitis, encephalitis, post-transplant lymphoproliferative disease (PTLD), and CNS lymphoma are associated with EBV infection although antiviral therapy was either ineffective [87] or successful [78,88] in such cases. Results on clinical efficacy are therefore inconsistent. In another study, co-administration of adjuvant interferon-beta after radiochemotherapy for 17 squamous cell carcinomas and nine undifferentiated carcinomas showed a 100% survival rate at 96 months [44]. It is known that adjuvant interferon-beta therapy in combination with a high dose-hyperfractionated radiation increases the efficacy of intensely modified radiation therapy.

Despite a vast number of reported cases, the role of infectious agents in the mechanisms of carcinogenesis demands further attention from clinicians. Statistically, it appears that infections are associated with the vast majority of brain and head and neck cancers, and it therefore seems likely that such infections may at the very least modulate cancer pathogenesis. However, it is unclear whether the infectious environment predisposes the patient to carcinogenesis by down-regulating the host immune system or whether cancer cells debilitate the cellular and immune response so that the infectious agents can easily overcome the defence mechanisms and evade the host cells. Nevertheless, the effectiveness of anti-virals, antibiotics, and therapeutic vaccines against onco-associated infections in cancer treatment has been reported in several studies. Based on the data reported in this review, we believe that combating pathogens could be a key treatment strategy to reduce or even prevent brain and head and neck malignancies.

## Abbreviations

CBP/p300: CREB binding protein/p300; 0CMV: Cytomegalovirus; COX-2: Cyclooxygenase 2; SV40: Simian virus 40; BKV: BK virus; JCV: John Cunningham virus; EBER: Epstein-Barr virus-encoded small RNA; EBNA: Epstein-Barr nuclear antigens; EBV: Epstein-Barr virus; EDNRB: Endothelin receptor type B; GBM: Glioblastoma multiforme; HCMV: Human cytomegalovirus; HNSCC: Head and neck squamous cell carcinoma; HPV: Human papilloma virus; IARC: International Agency for Research on Cancer; IgA: Immunoglobulin A; IL: Interleukin; LMP: Latent membrane proteins; MHC: Major histocompatibility complex; NPC: Nasopharyngeal carcinoma; OSCC: Oral squamous cell carcinoma; KS: Kaposi’s sarcoma; PGE2: Prostaglandin E2; PI3K/AKT: Phosphatidylinositol-3-kinase and protein kinase B; RASSF1A: Ras association domain family 1A; MAPK: Mitogen-activated protein kinase; β-FGF: β-fibroblast growth factor; PDGF: Platelet-derived growth factor; VEGF: Vascular endothelial growth factor; Tat: Trans-activator of transcription.

## Competing interests

The authors declare that they have no competing interests.

## Authors’ contributions

KA, AK, and YB performed the literature research and wrote the manuscript. All authors read and approved the final manuscript.
